# Thyroid dysfunction in patients with impaired glucose metabolism: 11 year follow up from the Tehran Thyroid Study

**DOI:** 10.1371/journal.pone.0184808

**Published:** 2017-10-03

**Authors:** M. Gholampour Dehaki, A. Amouzegar, H. Delshad, Y. Mehrabi, M. Tohidi, F. Azizi

**Affiliations:** 1 Endocrine Research Center, Research Institute for Endocrine Sciences, Shahid Beheshti University of Medical Sciences, Tehran, I.R. Iran; 2 Department of Internal Medicine, School of Medicine, Aja University of Medical Science, Tehran, I.R. Iran; 3 Obesity Research Center, Research Institute for Endocrine Sciences, Shahid Beheshti University of Medical Sciences, Tehran, I.R. Iran; 4 Department of Epidemiology, School of Health, Shahid Beheshti University of Medical Sciences, Tehran, I.R. Iran; 5 Prevention of Metabolic Disorders Research Center, Research Institute for Endocrine Sciences, Shahid Beheshti University of Medical Sciences, Tehran, I.R. Iran; The University of Tokyo, JAPAN

## Abstract

**Objectives:**

This study aimed to assess the prevalence and incidence and predictive factors of thyroid disorders (TD) in patients with impaired glucose metabolism.

**Methods:**

Prevalence of TD was calculated in patients with impaired glucose metabolism compared to healthy controls, aged over 30 years in phase 1 of the Tehran Thyroid Study (TTS). Follow up assessments were conducted every 3 yrs, after which incidence of TD was calculated and its correlations with age, sex, smoking, blood pressure, body mass index (BMI), thyroid peroxidase antibody (TPOAb), thyrotropin (TSH), insulin resistance index, triglycerides and cholesterol were assessed.

**Results:**

Incidence of TD among 435 diabetics, 286 prediabetics, and 989 healthy controls at baseline was 14, 18, and 21 per 1000 patients per year, respectively, being significantly lower in diabetics than that in healthy controls, a difference however that was not significant after adjusting for the variables mentioned (OR:0.64, 95% CI: 0.39–1.01). The incidence of TD in subjects with baseline serum TSH>1.94 mU/L or TPOAb≥40 IU/ml in all three groups was higher than that in patients with TSH≤1.94 mU/L or TPOAb<40 IU/ml, and remained significant after variable adjustment. Baseline TSH>1.94 mU/L was predictive of TD with 70% sensitivity and specificity. Baseline serum TSH (ROC area: 0.73, 95% CI: 0.68–0.77) had better predictive value than TPOAb (ROC area: 0.65, 95% CI: 0.61–0.69) for developing TD.

**Conclusion:**

Incidence of TD in type 2 diabetics or prediabetics is not higher than healthy controls. It is however necessary to conduct thyroid tests in patients with TPOAb≥40 IU/ml or TSH>1.94 mU/L.

## Introduction

Disorders of the thyroid gland and carbohydrate metabolism such as diabetes mellitus (DM), and prediabetes are among the most common endocrine disorders [1]. In a study by Wickham et al, the prevalence of thyroid disorders (TD) was reported to be 6.6% in the UK [2]. According to the first phase of Tehran Lipid and Glucose Study (TLGS), the prevalence of TD is around 14.5% in Iranians, aged over 20 years [3]. Different studies have reported prevalences between 1–7% for hypothyroidism and between 0.5–3.5% for hyperthyroidism in diabetic patients [4–7]. According to a study conducted by the World Health Organization (WHO), the prevalence of DM in 2000 was 2.2% and is estimated to reach 4.4% by 2030; in other words, the total number of diabetic patients will reach 366 million by 2030 from 171 million in 2000 [8]. The relationship between TD and diabetes has long been a subject of interest and many studies have investigated the correlation between type 1 diabetes and TD, yet literature on the association of type 2 diabetes, prediabetes, with TD is limited [9–19]. No documented evidence exists regarding the prevalence and incidence of TD in patients with impaired fasting glucose (IFG) or those with impaired glucose tolerance (IGT). Undetected thyroid disorders may compromise metabolic control of patients with diabetes, IGT, or IFG, and may increase the risk of cardiovascular diseases [20–23]. On the other hand, diabetes and prediabetes can affect thyroid tests [24]. Considering the fact that thyroid screening tests are currently recommended only for high-risk groups, i.e. infants, pregnant women and the elderly, conducting these tests in diabetics and prediabetics requires further investigation [25–29]. Extensive knowledge about the prevalence and the interaction between TD, and DM, and prediabetes is necessary in order to design and implement screening programs, and to come up with appropriate specific treatment protocol for patients suffering from these conditions. The goal of this study was therefore to assess the prevalence and incidence of TD and their predictive factors in diabetic and prediabetic patients.

## Methods

### Study population

The present study, conducted within the framework of the Tehran Thyroid Study (TTS), evaluated 5786 persons (2376 men and 3410 women), aged ≥20 years in the cross-sectional phase, implemented from March 1999 to December 2001. Study participants were followed every 3 years during phases 2, 3, 4 [30]. To determine the prevalence of TD (cross sectional), we reviewed the data from diabetic and prediabetic patients, aged over 30 years, participants of phase 1 of the TTS. Patients with a history of thyroidectomy or radioactive iodine intake of non-specific cause, those taking glucocorticoids or lithium, and pregnant women were excluded from this study. In order to determine the incidence rate (cohort), the inclusion criteria were diabetic, prediabetic or healthy subjects,aged over 30 yrs. who had participated in the phase 1 of the TTS and at least one of the other 3 phases. Prediabetic patients were included if they had remained prediabetic in at least 2 consecutive phases. Criteria for healthy controls required that they should not have developed diabetes or prediabetes in any of the phases. Excluded were those who had thyroid disease (clinical or subclinical hypothyroidism or hyperthyroidism) and those with a history of thyroid surgery or radioactive iodine intake, consumption of glucocorticoids, levothyroxine, methimazole, propylthiouracil, or lithium or were pregnant at the entry phase. The ethics committee of the Research Institute for Endocrine Sciences, Shahid Beheshti University of Medical Sciences approved the protocol for this study, and informed written consent was obtained from all subjects.

### Laboratory measurements

Fasting blood samples were drawn from all participants between 7:00 and 9:00 AM into vacutainer tubes at baseline and each reassessment. Free thyroxine (FT4) and thyroid stimulating hormone (TSH) were determined in serum samples stored at -70 C, by the electrochemiluminescence immunoasaay (ECLIA) method, using Roche Diagnostics kits & Roche/Hitachi Cobas e- 411 analyzer (GmbH, Mannheim, Germany). Lyophilized quality control material (Lyphochek Immunoassay plus Control, Bio-Rad Laboratories) was used to monitor accuracy of assay; the intra- and inter-assay coefficients of variants (CVs) were 1.3% and 3.7% for FT4 and 1.5% and 4.5% for TSH determinations respectively. Thyroid peroxidase antibody (TPO Ab) was assayed by immune-enzymometric assay (IEMA) using related kit (Monobind, Costa Mesa, CA, USA) and the Sunrise ELISA reader (Tecan Co., Salzburg, Austria); intra- and inter-assay CVs were 3.9% and 4.7%, respectively. All biochemical tests for glucose, triglyceride and cholesterol assessments were performed on the day of sampling, using commercial kits (Pars Azmon Inc., Iran) by the Selectra 2 auto-analyser (Vital Scientific, Spankeren, The Netherlands); intra- and inter-assay CVs were < 2.3% for glucose and <2.1% for triglycerides, and <1.9% for cholesterol serum level.

### Definitions and terms

Based on the TTS, the 2.5^th^ and 97.5^th^ percentiles of normalized TSH were 0.32 mU/L and 5.06 mU/L, and of FT4 were 0.91 ng/ml and 1.55 ng/ml respectively [31]. Thyroid dysfunction was defined as clinical hypothyroidism with FT4<0.91 ng/dl and TSH>5.06 mU/L or receiving levothyroxine due to clinical hypothyroidism or TSH>10 mU/L; clinical hyperthyroidism with FT4>1.55 ng/dl and TSH<0.32 mU/L or receiving methimazole or propylthiouracil; subclinical hypothyroidism with 10>TSH>5.06 mU/L and 0.91≤FT4≤1.55; subclinical hyperthyroidism with TSH<0.32 mU/L and 0.91≤FT4≤1.55 ng/dl. Diabetes mellitus type 2 was defined with fasting blood glucose (FBS) ≥126 mg/dl or two-hour blood glucose ≥200 mg/dl during an oral glucose tolerance test (OGTT) with 75 gr glucose or taking antidiabetic drugs; prediabetes with 100≤FBS<126 mg/dl or 140≤ Two-hour BS<200 mg/dl during an OGTT with 75 gr glucose.

### Statistical analysis

Continuous data were analyzed using mean ± standard deviation (SD). If the distribution was not normal, data were expressed as median and percentiles. Categorical data were reported as percentages. Data analysis was carried out using the SPSS program (SPSS Inc., Chicago, IL, USA; Version 15) and P-values < 0.05 were considered statistically significant. Logistic regression was used to evaluate the effect of risk factors and to calculate odds ratios. Confounding variables were adjusted for each mentioned variable. ANOVA was used for the comparison of thyroid function variables including FT4 and TSH. To assess the incidence rate of TD during phases 1–4, the follow up period for each patient was calculated and the annual incidence rate of TD based on the patient/year was calculated with STATA software (Version 12). Kaplan Meier analysis was used to analyze the survival rate until the development of TD in different subgroups. ROC curve with STATA software was used to assess the cut-off points for baseline serum TSH and TPOAb for the prediction of TD and comparison between groups.

## Results

### Prevalence of thyroid disorder

Of 3,276 individuals initially enrolled, after applying the inclusion and exclusion criteria, 420 diabetics, 608 prediabetics and 2,104 healthy controls aged ≥ 30 years in phase one of the TTS were evaluated. Demographic data are summarized in Table 1. The prevalence of TD was 18.9, 19.3 and 13.5% in the three groups of diabetics, prediabetics, and healthy controls, respectively (P = 0.01). However, after using logistic regression to adjust for age, sex, smoking, blood pressure, body mass index (BMI), thyroid peroxidase antibody (TPOAb), thyrotropin (TSH), insulin resistance index, triglycerides and cholesterol, no significant difference was found between the three groups. Among the patients with newly diagnosed DM (comprising 46.9% of all diabetic patients) the prevalence of TD was 18%, compared to 19.9% in the previously diagnosed cases (P = 0.7). Separate assessment of TD after adjusting for above mentioned variables revealed that the prevalence of subclinical hyperthyroidism was significantly higher in diabetics compared to healthy controls (P = 0.02). The prevalence of clinical hypothyroidism was lower in prediabetics compared to healthy controls (P = 0.04). No other significant differences in prevalence of TD were noted among different groups (Table 2). After adjusting for the above variables, the prevalence of TD was significantly higher in female controls and prediabetics, compared to males (P = 0.008 and P<0.001, respectively); however, no such difference was found between diabetic males and females. The prevalence of TD was higher only in prediabetic patients aged > 50 yrs, compared to those <50 yrs (P = 0.02). The prevalence of TD was higher in subjects with TPOAb≥40 IU/ml, compared to those with TPOAb<40 IU/ml among the three groups (P = 0.001, P<0.001 and P = 0.008).

**Table 1:** Basal characteristics of the diabetic, prediabetic and healthy groups.

Characteristics	Prediabetic (n = 420)	Diabetic (n = 608)	Healthy (n = 2104)	P Value
Age,(years)*	54.1±10.6	49.9±11.4	42.4±10.4	0.01
Females (%)	56.3	53.3	58.5	0.27
BMI (Kg/m2)*	28.8±4.3	28.6±4	26.5±4	0.70
Weight (kg)*	74.6±12.8	74.6±11.5	69.8±11.4	0.09
SBP (mmHg)*	131±20.6	125±18.7	113±15.3	<0.01
DBP (mmHg)*	81.7±10.5	81±10.2	75.2±9.8	0.90
FBS (mg/dl)*	161±57	101±9.1	86±6.6	<0.01
Serum TSH (mU/l)†	1.46(0.9–2.1)	1.51(1.02–2.49)	1.59(1.02–2.49)	0.02
SerumFT4 (ng/dl)*	1.21±0.16	1.18±0.17	1.21±0.17	0.51
TPO Ab (IU/ml)†	5.3(3.3–9.8)	5.6(3.2–9.7)	4.8(3–10.1)	0.21
Smoking positive (%)	9.8	8.2	13.7	0.01
Cholesterol (mg/dl)*	226±49	222±43	200±40	<0.01
Triglycerides (mg/dl)†	207(150–289)	181(120–247)	126(90–176)	<0.01
Insulin(mU/l)†	9.2(6.2–12.9)	9.0(6.2–12.2)	4.8 (6.6–8.9)	<0.01
HOMA-IR†	2.3 (3.3–9.8)	2.2 (1.5–3.0)	1.4(0.9–1.9)	<0.01

**Abbreviations**: BMI, body mass index(calculated as weight in kilograms divided by height in meters squared);SBP, systolic blood pressure; DBP, diastolic blood pressure; FBS, fasting blood sugar

*Values are presented as mean (SD) for continuous parameters

^†^ Median (IQR) for variables without normal distribution.

**Table 2:** Comparison of the prevalence of thyroid disease among diabetic, prediabetic and healthy groups*.

	Comparison	OR	95% CI	P Value
Thyroid dysfunction	PreDM vs NL	1.03	0.77–1.38	0.80
	DM vs NL	1.32	0.93–1.87	0.11
Overt hypothyroidism	PreDM vs NL	0.42	0.18–0.98	0.04
	DM vs NL	0.93	0.41–2.10	0.87
Overt hyperthyroidism	PreDM vs NL	1.03	0.50–2.15	0.92
	DM vs NL	0.39	0.12–1.27	0.12
Subclinical hypothyroidism	PreDM vs NL	1.41	0.91–2.09	0.12
	DM vs NL	1.45	0.89–2.35	0.18
Subclinical hyperthyroidism	PreDM vs NL	1.01	0.61–1.68	0.95
	DM vs NL	1.84	1.06–3.18	0.02

**Abbrevtionias**: PreDM, Prediabetes; DM, Diabetes mellitus; NL, Normal

*Adjusted with logistic regression for age, sex, smoking, blood pressure, body mass index (BMI), thyroid peroxidase antibody (TPOAb), insulin resistance index, triglycerides and cholesterol

### Incidence of thyroid disorder

**Fig 1:**
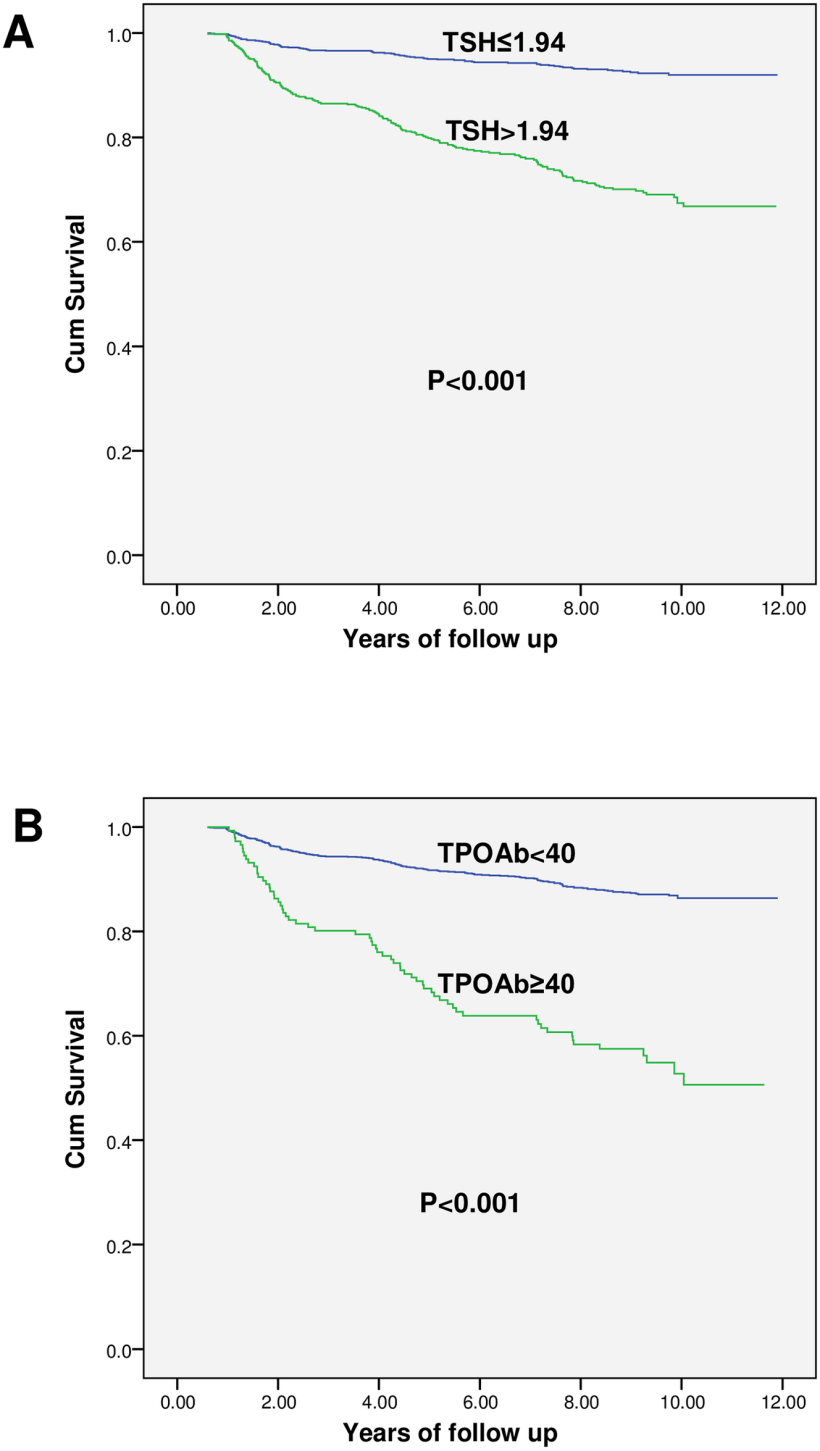
Kaplan Meier curve for the survival without thyroid disease, based on serum TPOAb and normal baseline TSH.

**Fig 2:**
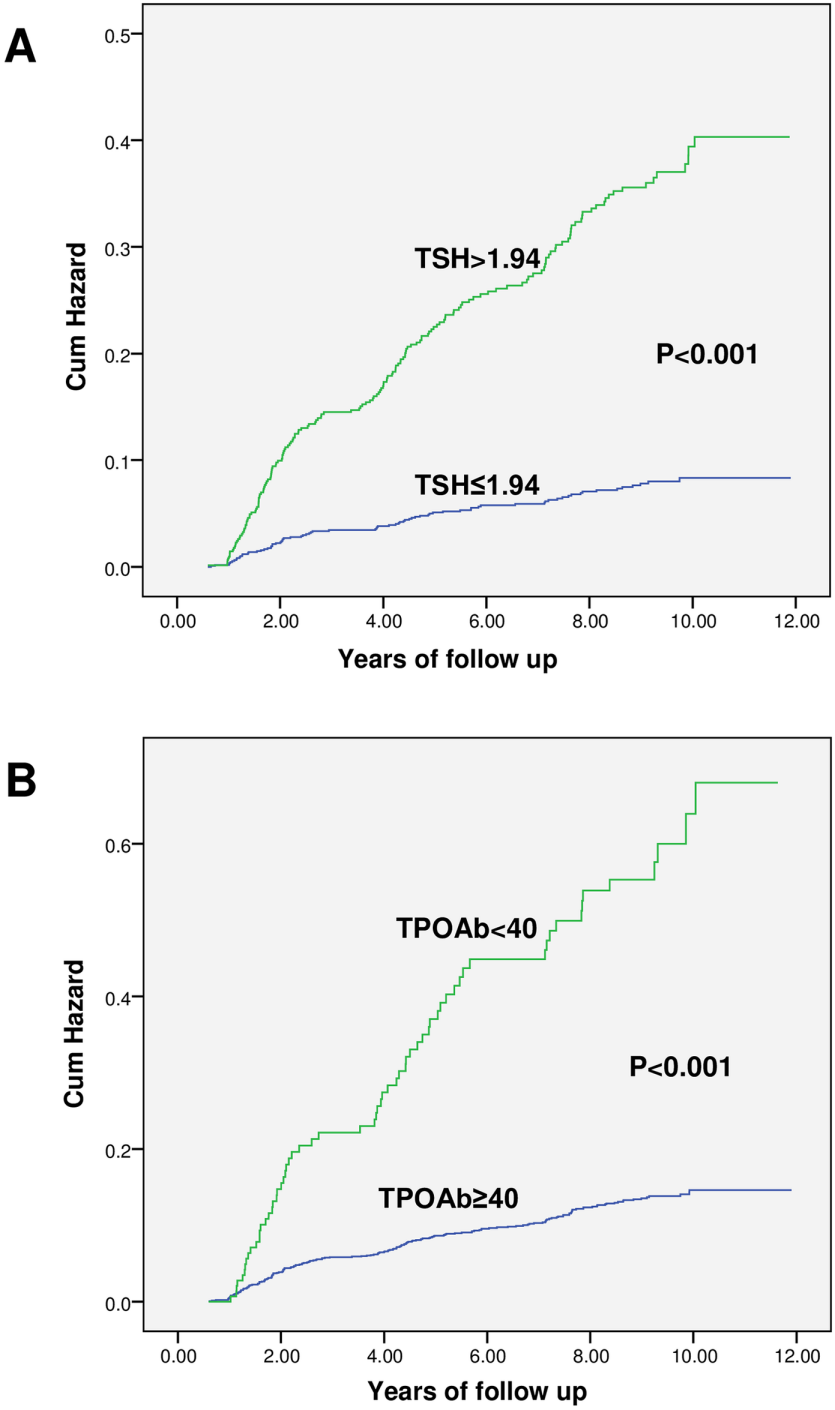
Kaplan Meier curve for the cumulative incidence hazard of thyroid disease according to serum TPOAb and normal baseline TSH.

**Fig 3:**
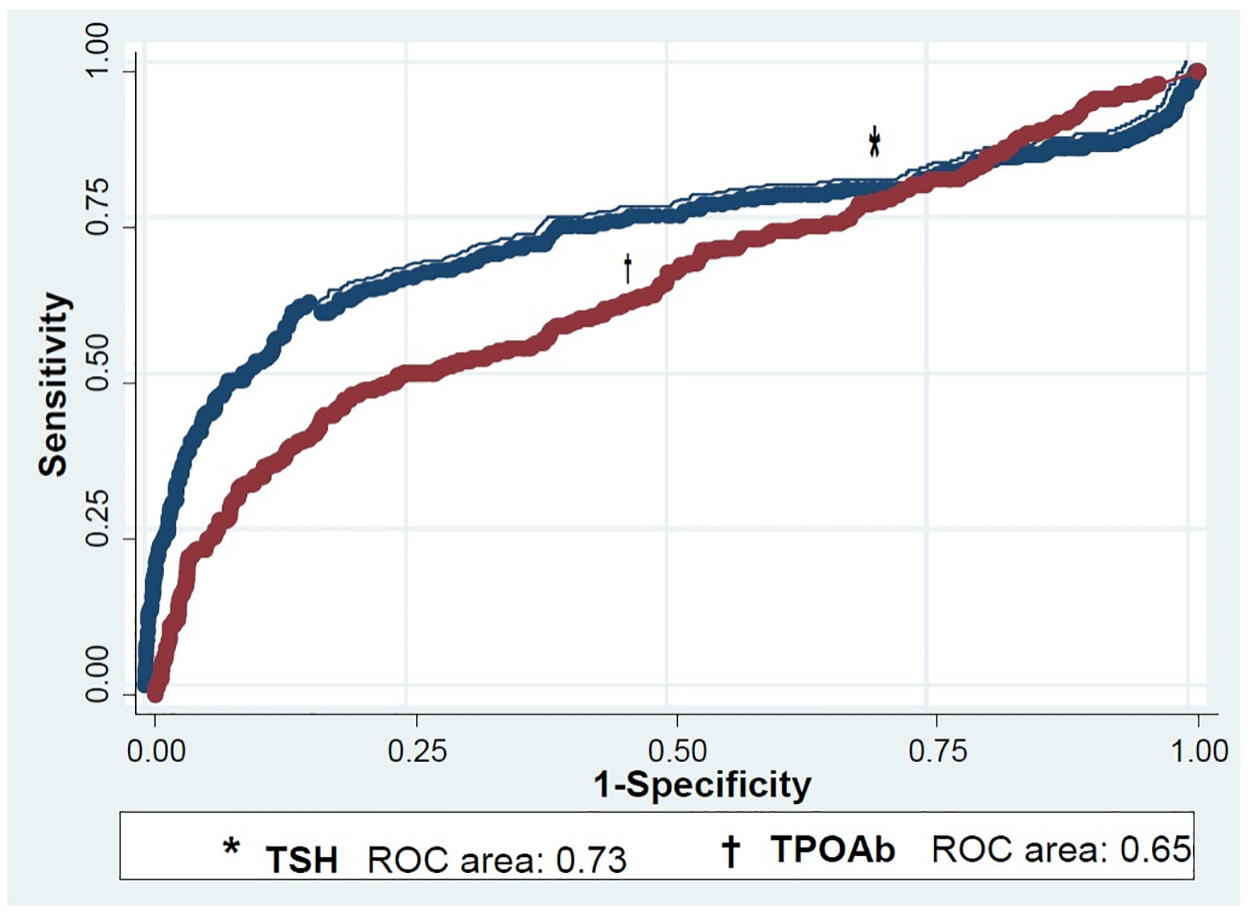
ROC curve for the comparison of baseline TSH with TPOAb as the predictive factors for the incidence of thyroid disease

After applying the inclusion and exclusion criteria, 989 healthy controls, 286 prediabetic patients, and 435 diabetics were selected from the phase one of the TTS to calculate the incidence of TD (Table 3). In general, the incidence of TD per 1000 person—years was 14, 18 and 21 in diabetics, prediabetics and healthy controls, respectively. The incidence of TD was significantly lower in diabetic patients than in healthy controls and remained significant after adjusting for age, sex, smoking, blood pressure, BMI, TPOAb, insulin resistance index, triglycerides and cholesterol (except for TSH) using logistic regression [Odd Ratio (OR),0.59; 95% confidence interval (CI), 0.36–0.95]. However, after adjusting for all variables, including baseline TSH, no significant difference was found for incidence of TD between groups (OR, 0.64; 95% CI, 0.39–1.01); however incidence of subclinical hypothyroidism was significantly lower in diabetics compared to healthy controls (OR, 0.47; 95% CI; 0.25–0.89) (Table 4). Compared to previously diagnosed cases, the incidence of TD in newly diagnosed diabetics was 13% versus 10.3%, not significant (P = 0.44), even after adjusting for other variables, including baseline TSH (OR, 1.13; 95% CI, 0.55–2.29). The incidence of TD was only higher in prediabetic and healthy females compared to males (P<0.001, and P = 0.01, respectively) and there was no difference between diabetic males and females (P = 0.1) (Table 5). The prevalence of baseline TPOAb>40 IU/ml was 25% in subjects with TD, 24% in diabetics, 17.5 in prediabetics, and 28.5% in healthy controls. However, the overall prevalence of TPOAb>40 IU/ml was 8.9%, being 7.2, 7.1, and 9.8% in diabetics, prediabetics and healthy controls, respectively. In general, incidence of TD in subjects with TPOAb≥40 IU/ml in all three groups was significantly higher than those with TPOAb<40 IU/ml and remained significant after adjusting for above variables (P<0.001, P = 0.02 and P = 0.001 for diabetics, prediabetics, and healthy controls, respectively). Also, after adjusting for possible confounders, the incidence of TD in all three groups with baseline TSH within the reference value, but >1.94 mU/L, was higher than that in subjects with baseline TSH≤1.94 mU/L (P = 0.001); in other words, subjects with TSH>1.94 mU/L (but within the reference limit) diabetic, prediabetic, and healthy control groups had 3.4, 5, and 4.7 times higher risks of developing TD compared to their counterparts with baseline TSH≤1.94 mU/L. In general, baseline TSH>1.94 mU/L is predictive of TD with sensitivity and specificity of 70%. We found no significant difference in the cut off points among the three groups. The predictive value of serum TSH>2.27 mU/L for TD was greater in females than in males with 68% sensitivity and 87% specificity. "Figs 1 and 2" compare the survival without TD and the cumulative risk of TD based on normal baseline TSH and serum TPOAb. As seen in" Fig 3", according to the ROC curve, serum TSH (ROC area: 73.0, 95% CI: 68.0–77.0) had a higher predictive value than TPOAb (ROC area: 0.65, 95% CI: 0.61–0.69) for TD (P = 0.001)

**Table 3:** Demographic characteristics of the diabetic, prediabetic and healthy groups for estimation of the thyroid disease incidence.

Characteristics	Diabetic(n = 435)	Prediabetic (n = 286)	Healthy (n = 989)	P Value
Age (years)*	54.11±0.6	49.9±11.4	42.4±10.4	0.01
Female (%)	56.3	53.3	58.5	0.27
BMI (Kg/m2)*	28.8±4.3	28.6±4	26.5±4	0.70
Weight (kg)*	74.6±12.8	74.6±11.5	69.8±11.4	0.09
SBP (mmHg)*	131±20.6	125±18.7	113±15.3	<0.01
DBP (mmHg)*	81.7±10.5	81±10.2	75.2±9.8	0.90
FBS (mg/dl)*	161.1±57	101±9.1	86±6.6	<0. 01
Serum TSH (mU/l)†	1.46(0.9–2.1)	1.51(1.022.49)	1.59 (1.022.69)	0.02
SerumFT4 (ng/dl)*	1.2±10.16	1.18±0.17	1.21±0.17	0.08
TPO Ab (IU/ml) †	5.3(3.3–9.8)	5.6(3.2–9.7)	4.8(3–10.1)	0.21
Smoking positive (%)	9.8	8.2	13.7	0.01
Cholesterol (mg/dl)*	226±49	222±43	200±40	<0.01
Triglyceride (mg/dl)†	207(150–289)	181(120–247)	126(90176)	<0.01
Insulin (mU/l)†	9.2(6.2–12.9)	9.0(6.2–12.2)	4.8(6.6–8.9)	<0.01
HOMA-IR †	2.3(3.3–9.8)	2.2(1.5–3.0)	1.4(0.9–1.9)	<0.01

**Abbreviations**: BMI, body mass index(calculated as weight in kilograms divided by height in meters squared);SBP, systolic blood pressure; DBP, diastolic blood pressure; FBS, fasting blood sugar.

*Values are presented as mean (SD) for continuous parameters

^†^ Median (IQR) for variables without normal distribution.

**Table 4:** Comparison of the incidence of thyroid disease in the diabetic, prediabetic and healthy.

Conditions	Comparison	OR; 95%CI		P-value	
		Model 1	Model 2	Model 1	Model 2
Thyroid dysfunction	Pre DM vs NL	0.89 (0.57–1.38)	0.91 (0.58–1.42)	0.60	0.69
	DM vs NL	0.59 (0.36–0.95)	0.64 (0.39–1.0)	0.03	0.07
Overt hypothyroidism	PreDM vs NL	0.83 (0.29–2.38)	0.72 (0.23–2.2)	0.74	0.58
	DM vs NL	0.71 (0.24–2.07)	0.96 (0.32–2.8)	0.54	0.59
Overt hyperthyroi1dism	PreDM vs NL	1.88 (0.07–6.94)	0.69 (0.07–6.6)	0.49	0.75
	DM VS NL	0.99 (0.31–11)	1.89(0.30–11.6)	0.99	0.49
Subclinical hypothyroidism	PreDM vs NL	0.84 (0.51–1.38)	0.89 (0.51–1.56)	0.49	0.69
	DM vs NL	0.38 (0.21–0.69)	0.47 (0.25–0.89)	0.001	0.02
Subclinical hyperthyroidism	PreDM vs NL	1.03 (0.34–3.13)	0.99 (0.31–1.32)	0.95	0.99
	DM vs NL	1.64 (0.59–4.56)	1.38 (0.48–3.9)	0.33	0.54

Model 1: Adjusted with logistic regression for age, sex, smoking, blood pressure, body mass index (BMI), thyroid peroxidase antibody (TPOAb), insulin resistance index, triglycerides and cholesterol

Model 2: Adjusted with logistic regression for variables like Model 1 plus base TSH

**Table 5:** The effect of several co-variants on the incidence of thyroid dysfunction in the diabetic, prediabetic and healthy groups, based on multivariant logistic regression analysis.

variable	Incidence of thyroid dysfunction		
	Diabetic	Prediabetic	Healthy
Sex Females vs males	1.63(0.81–3.28) *	2.80(1.18–6.61)	2.06(1.32–3.21)
BMI(Kg/m2) ≥30 vs <30	0.53(0.25–1.09)	0.72(0.32–1.59)	0.90(0.56–1.43)
TPO Ab(IU/ml) ≥40 vs <40	5.6(2.41–13.1)	3.32(1.16–9.5)	5.66(3.52–9.10)
Age, years ≥50 vs <50	0.88(0.43–1.77)	1.25(0.57–2.76)	0.72(0.44–1.17)
Smoking Positive VS negative	0.81(0.22–2.91)	1.8(0.21–5.42)	0.75(0.38–1.48)
HOMA-IR ≥2.5 vs <2.5	1.67(0.77–3.64)	1.33(0.62–2.82)	0.99(0.53–1.85)
SBP(mmHg)	0.99(0.97–1.00)	0.99(097–1.01)	1.00(0.99–1.02)
Cholestrol(mg/dl)	0.99 (0.97–1.00)	1.00(0.99–1.01)	1.00(1.00–1.02)
TSH (mU/L) >1.94 vs ≤1.94	3.43(1.7–6.7)	5.08 (2.3–11.0)	4.75(3.1–7.1)

**Abbreviations**: BMI, body mass index(calculated as weight in kilograms divided by height in meters squared); SBP, systolic blood pressure; DBP, diastolic blood pressure; FBS, fasting blood sugar

*Odd Ratio (95% Confidence Interval)

## Discussion

In this study the prevalence of TD was 18.9, 19.3 and 13.5% and the incidence of TD per 1000 person—years was 14, 18 and 21 in the diabetic, prediabetic, and healthy control groups, respectively. The incidence of TD in subjects with baseline serum TSH>1.94 mU/L but within the reference range (0.32–5.06) or TPOAb≥40 IU/ml in all three groups was higher than that in patients with TSH≤1.94 mU/L or TPOAb<40 IU/ml, and remained significant after variable adjustment. Baseline TSH>1.94 mU/L was predictive of TD with 70% sensitivity and specificity. Baseline serum TSH (ROC area: 0.73, 95% CI: 0.68–0.77) had better predictive value than TPOAb (ROC area: 0.65, 95% CI: 0.61–0.69) for developing TD.

In a study conducted in Spain on 318 type 2 diabetic patients aged, 29–89 years old, the prevalence of TD was found to be 32.4% [17]. The prevalence rate varies from 10.8–45% in other studies [12, 16–19, 29]. In our study, no significant difference was observed in prevalence of TD among the three groups of diabetics, prediabetics and healthy controls. However, in a study conducted in Jordan, prevalence of TD in 908 diabetics was reported to be 12.5%, which was significantly higher compared to 6.6% prevalence rate in a control group of 304 healthy subjects, it should be noted that this study was not conducted on the general population, and the number of controls was less than half that of the diabetic patients; moreover, patients who had undergone thyroidectomy, or were receiving radioactive iodine were not excluded from the hypothyroidism group [29]. The prevalence of TD in the aforementioned study was higher in diabetic males compared to that in females, findings in contrast to the results of several other studies reporting a higher prevalence among females (10.2% versus 6.9% in a study from Scotland and 39.9% versus 22% in a study in Spain)[12, 17, 29]. Papazafiropoulou et al., and Al-Geffari et al. also reported female sex to be a predictive factor for TD in diabetics [19, 32]. In our study, the prevalence and the incidence of TD were higher only in healthy controls and prediabetic females, compared to males, and no such difference was noted in the diabetic group. Based on studies by Canaris and Hollowell, being elderly is associated with an increased prevalence of TD [6, 7]. However, in our study, after adjusting for possible confounders no difference was noted in the prevalence of TD between diabetics and healthy controls, aged below or over 50 yrs. The prevalence of TD, however, was significantly higher in prediabetics over 50 yrs. Similar to many previous studies, our study showed that subclinical hypothyroidism, and clinical hyperthyroidism were the most and the least common TD in diabetics, respectively [17–19, 25]; however, the rates observed for these disorders in our study were different from those in similar studies which could be attributed to the different definitions used for these disorders in other studies, the difference in the reference limits for TSH and FT4, the amount of iodine intake, and the sensitivity of assays used, as well as ethnic and geographical differences. In our study, the mean incidence of TD was 1.4, 1.8, and 2.1 per 1000 person—years in diabetics, prediabetics and healthy controls, respectively in a mean follow up period of 8.6 years. In a study done in Spain, the incidence of TD over a 5-year follow up was reported to be 7.1% in diabetics and 3.8% in controls; however after adjusting for confounders this difference was not statistically significant [33]. In another study from Scotland, the incidence of TD over a one-year follow up of 1,310 patients with type 1 and type 2 diabetes was 6.8% [12], in this study, the incidence of clinical hyper- and hypothyroidism in diabetics was higher than that in our study; however, unlike the cross sectional nature of our study, their study included only patients presenting to a diabetes clinic and their follow up period was only one year. In a study conducted in the UK on a target population of type 1 and type 2 diabetics, with an average follow up of 6.1 years, the prevalence of TD in euthyroid diabetic patients with baseline thyrotropin >1.53 mU/L was seven times higher than that in subjects with thyrotropin<1.53mU/L, the reference range for was 0.15–3.5 mU/L [25]. Vanderpump et al. reported positive thyroid antibodies and baseline TSH>2mU/L as two risk factors for developing TD in the general public [26]. In our study, baseline TSH and positive TPOAb were predictive factors for TDs in all groups, and the risk of TD in diabetics, prediabetics, and controls with baseline TSH>1.94 mU/L (but within the reference limit) was 3.4, 5 and 4.7 times that in individuals with baseline TSH<1.94mU/L; these rates were 5.6, 3.3, and 5.6 times higher in subjects with TPOAb>40IU/ml compared to those with TPOAb<40IU/ml. Therefore, considering the overall 36% prevalence of subjects with TSH>1.94 mU/L, and 9.8% overall prevalence of subjects with TPOAb>40 IU/ml, conducting screening tests with shorter intervals (probably annually) seems rational and cost-effective in these subjects.

Of the limitations to our study, one is not knowing the adequacy of the subjects’ daily iodine intake (i.e not measuring the amount of iodine excreted in the urine in 24-h); however since this study was performed in an iodine rich area, this shortcoming is unlikely to have interfered with the results. Another limitation was that the thyroid tests in each phase and for each subject were conducted only once, while in order to diagnose TD with certainty, these tests should be repeated within 1–4 months of the first test; the intervals between thyroid tests were also too long; therefore the exact time of the occurrence of TD or the change in thyroid function could not be determined. Moreover, since Glutamic Acid Decaboylase Antibody (GADAb) was not checked, cases of Latent Auto-immune Diabetes in Adults (LADA) and type 2 diabetes were not differentiated. However, in our study, no significant difference was noted in the prevalence and the incidence of TD between the newly diagnosed diabetics and those with long standing diabetes. Another limitation of our study was not ascertaining the reason why some patients took levothyroxine. However, considering our large sample size and the small number of such patients, this shortcoming would probably not have affected our results. Also, in our study, consumption of methimazole and propylthiouracil was considered a sign of clinical hyperthyroidism, which could have lead to over-estimation of the prevalence of clinical hyperthyroidism and underestimation of subclinical hyperthyroidism. However, due to our large sample size and small number of these patients, this shortcoming is also not likely to have compromised our results. As for our strengths, a large sample size representative of the urban population of Tehran, simultaneous conduction of all thyroid tests at the same time in each patient, using same assay methods for each test, and a long follow up period are among the main strengths of our study. Moreover, our study is the first to be conducted to determine the prevalence and incidence of TD in prediabetics. A cohort study with a larger sample size and longer follow up period is recommended to focus on the assessment of TD in diabetics and prediabetics to gain more insight into this topic. It is concluded that the incidence and the prevalence of TD in type 2 diabetics and prediabetics are not higher than those in healthy controls. Thus, conducting screening tests is not recommended in all patients, except in those with TPOAb≥40IU/ml or TSH>1.94mU/L.
